# Set3 contributes to heterochromatin integrity by promoting transcription of subunits of Clr4-Rik1-Cul4 histone methyltransferase complex in fission yeast

**DOI:** 10.1038/srep31752

**Published:** 2016-08-19

**Authors:** Yao Yu, Huan Zhou, Xiaolong Deng, Wenchao Wang, Hong Lu

**Affiliations:** 1 State Key Laboratory of Genetic Engineering, School of Life Sciences, Fudan University, Shanghai, 200438, China; 2 Shanghai Engineering Research Center Of Industrial Microorganisms, Shanghai, 200438, China; 3 Shanghai Collaborative Innovation Center for Biomanufacturing Technology, Shanghai, 200237, China

## Abstract

Heterochromatin formation in fission yeast depends on RNAi machinery and histone-modifying enzymes. One of the key histone-modifying complexes is Clr4-Rik1-Cul4 methyltransferase complex (CLRC), which mediates histone H3K9 methylation, a hallmark for heterochromatin. CLRC is composed of the Clr4 histone methyltransferase, Rik1, Raf1, Raf2 and Pcu4. However, transcriptional regulation of the CLRC subunits is not well understood. In this study, we identified Set3, a core subunit of the Set3/Hos2 histone deacetylase complex (Set3C), as a contributor to the integrity and silencing of heterochromatin at centromeres, telomeres and silent mating-type locus. This novel role of Set3 relies on its PHD finger, but is independent of deacetylase activity or structural integrity of Set3C. Set3 is not located to the centromeric region. Instead, Set3 is targeted to the promoters of *clr4*^+^ and *rik1*^+^, probably through its PHD finger. Set3 promotes transcription of *clr4*^+^ and *rik1*^+^. Consistently, the protein levels of Clr4 and Rik1 were reduced in the *set3*Δ mutant. The heterochromatin silencing defect in the *set3*Δ mutant could be rescued by overexpressing of *clr4*^+^ or *rik1*^+^. Our study suggests transcriptional activation of essential heterochromatin factors underlies the tight regulation of heterochromatin integrity.

Heterochromatin represents a heavily condensed and repressive form of chromatin. It plays crucial functions in chromatin segregation, genomic transcription and epigenetic gene silencing during mitosis and meiosis, thus maintaining genomic stability. In fission yeast, constitutive heterochromatin is found mainly at telomeres, the silent mating-type locus, and centromeres^1^. The assembly of pericentromeric heterochromatin is tightly regulated by the interplay of RNAi machineries and enzymes that modify histones. During S phase, nascent transcripts arising from the outer repeats (divided into elements known as *dh* and *dg*) of centromeric region are transcribed by an RNA-directed RNA polymerase complex (RDRC) into double-stranded RNA (dsRNA)^2^,^3^. dsRNAs are processed into siRNAs by Dicer (Dcr1) and delivered to a Argonaute chaperone complex (ARC), whereas siRNAs are loaded onto Argonaute (Ago1)^4^. Ago1 is assembled with Tas3 and Chp1 into an RNA-induced transcriptional silencing complex (RITS)^5^. siRNAs guide RITS to target nascent transcripts by sequence complementarity, and the transcripts are sliced by Ago1 to achieve transcriptional silencing^6^. Transcript-bound RITS recruits RDRC to produce more dsRNA and siRNA^7^. The self-reinforcing loop of RNAi is strengthened by histone-modifying enzymes, mainly through the connections between RITS and Clr4-Rik1-Cul4 methyltransferase complex (CLRC). Chromatin-bound-RITS recruits CLRC to the centromeric repeats through the linker protein Stc1^8^. Clr4, the catalytic subunit of CLRC, methylates lysine 9 of histone H3 (H3K9me)^9^. H3K9me is bound by Chp1, which stabilizes the association between RITS and chromatin^10^. H3K9me also provides binding sites for the other heterochromatin components including Swi6, Chp2, histone deacetylase complex SHREC, and Clr4 itself to result in the establishment and maintenance of heterochromatin^10^,^11^,^12^,^13^.

CLRC consists of Clr4, the β-propeller protein Rik1, the cullin protein Pcu4 (also known as Cul4), the WD-40 protein Raf1 (also known as Dos1/Clr8/Cmc1) and the Zn-finger protein Raf2 (also known as Dos2/Clr7/Cmc2)^14^,^15^,^16^,^17^,^18^. Not only Clr4, but all other subunits of CLRC are essential for the heterochromatin formation^19^. Rik1 is thought to act upstream of Clr4, and to help recruit CLRC and the RNAi machinery to chromatin^15^,^20^. Pcu4 supports the putative E3 ubiquitin ligase activity of CLRC, probably by targeting substrates essential for heterochromatin assembly^17^. Raf1 and Raf2 are required for the localization of Swi6, a protein crucial for organization of higher order heterochromatin^16^. Compared to the physical interactions between CLRC subunits and other heterochromatin components, the transcriptional regulation of CLRC subunits is less well understood.

Set3 was first characterized in budding yeast by its feature of containing PHD finger and SET domain^21^. The combination of both domains is a characteristic displayed by a group of trx-G proteins, including histone methyltransferase Trx and Ash1^22^. However, no methyltransferase activity has been reported for Set3. In budding yeast, Set3, Sif2, Snt1 and Hos2 forms the functional core of a histone deacetylase complex named Set3/Hos2 Complex (Set3C), in which Hos2 harbors deacetylase activity^21^. The composition of core Set3C is conserved in *Schizosaccharomyces pombe* and *Candida albicans*, and closely resembles the components of metazoan NCoR/SMRT corepressor complexes^23^,^24^,^25^. In budding yeast, Set3C is predominantly recruited to the 5′ transcribed region of genes to reduce the histone acetylation level^26^. The recruitment of Set3C is possibly mediated by the recognition of H3K4me2 by the PHD finger of Set3, or by the interaction between Set3C and Ser5-phosphorylated RNA polymerase II^26^,^27^. Set3C plays both repressive and activating roles in transcription, depending on the context of the region to which it is recruited^28^. Although the roles of Set3 in transcriptional regulation were reported or suggested in the context of Set3C, it is unclear whether Set3 can regulate transcription independent of Set3C. Here, we show that Set3 contributes to the integrity of heterochromatin by promoting the transcription of Clr4 and Rik1, two key subunits of CLRC. Unexpectedly, this role of Set3 is independent of Set3C, indicating a novel way of Set3-mediated transcriptional regulation.

## Results

### Set3 contributes to the integrity of constitutive heterochromatin

**Figure 1:**
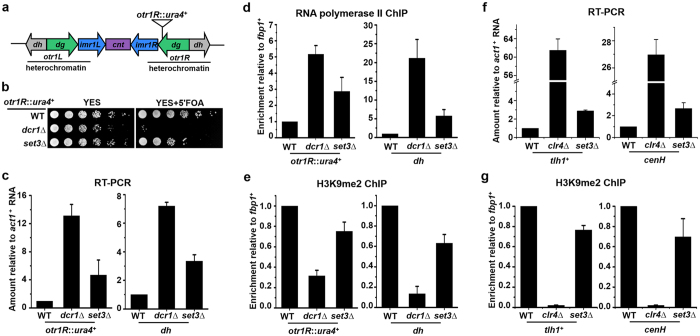
Set3 contributes to the integrity of heterochromatin. (**a**) A schematic representation of centromere 1 and the position of a inserted marker gene (*otr1R::ura4*). The pericentromeric heterochromatin region is indicated. (**b**) A fivefold serial dilution assay to examine the silencing of *otr1R::ura4*. Wild-type (WT) cells with silenced *ura4*^+^ grow normally on medium containing 5′-FOA, while loss of silencing kills cells on 5′-FOA. (**c**) RT-PCR analysis of *otr1R::ura4*^+^ and pericentromeric repeat (*dh*) RNA levels relative to a control *act1*^+^. The relative level in WT cells was arbitrarily designated as 1. Each column shown in (**c**) and below represents the mean ± s.d. from three biological repeats. (**d,e**) ChIP analysis of RNA Polymerase II (**d**) and H3K9me2 (**e**) at *otr1R::ura4* and *dh* relative to *fbp1*^+^. Relative enrichment in WT cells was arbitrarily designated as 1. (**f**) RT-PCR analysis of *tlh1*^+^ and *cenH* RNA levels relative to a control *act1*^+^. (**g**) ChIP analysis of H3K9me2 at *tlh1*^+^ and *cenH* relative to *fbp1*^+^.

To dissect the mechanism of heterochromatin assembly, we carried out a genetic screen for mutants that displayed a defect in centromeric silencing in fission yeast. We used a parental strain in which the native *ura4*^+^ gene was deleted and a *ura4*^+^ marker gene was inserted into the outermost (*otr*) pericentromeric heterochromatin of chromosome 1 (*otr1R*::*ura4*^+^) (Fig. 1a)^29^. Suppression of the *ura4*^+^ marker gene by heterochromatin results in normal growth of the cells on medium containing the counterselective drug 5-fluoroorotic acid (5′-FOA), which is toxic to cells expressing *ura4*^+^. Once heterochromatin is disrupted by a mutation, the expression of *ura4*^+^ is increased and mutant cells are killed by 5′-FOA. As shown in Fig. 1b, deletion of *set3*^+^ (*set3*∆) in this strain derepressed the *ura4*^+^ gene, and that resulted in relatively poor growth on 5′-FOA containing medium compared to wild type (WT) cells. The phenotype of *set3*∆ mutant was similar to that of cells lacking an essential component of the RNAi machinery (*dcr1*∆), but to a much less extent. Consistently, the transcripts from the *otr1R*::*ura4*^+^ and endogenous pericentromeric repeat (*dh*), and the occupancy of RNA polymerase II (RNA Pol II) at both loci increased slightly in *set3*Δ cells (Fig. 1c,d). H3K9 methylation is a hallmark of constitutive heterochromatin in most eukaryotes. *set3*Δ cells exhibited reduced levels of H3K9 dimethylation (H3K9me2) at *otr1R*::*ura4*^+^ and *dh*, indicating impaired integrity of pericentromeric heterochromatin. Nevertheless, the drop of H3K9me2 in *set3*Δ cells was not dramatic as that in *dcr1*∆ mutant (Fig. 1e). The integrity of heterochromatin at telomeres and silent mating-type locus was also investigated. As shown in Fig. 1f, the transcription of *tlh1*^+^, a gene embedded in the subtelomeric region^30^, and that of *cenH*, an element present at the silent mating-type locus^31^, increased slightly in *set3*Δ cells. Amounts of H3K9me2 at both loci decreased in *set3*Δ mutant compared with WT cells (Fig. 1g). However, the defects of heterochromatin at both loci in *set3*Δ mutant are much weaker than those observed in a *clr4*Δ mutant (Fig. 1f,g). Thus, results suggest Set3 is not an essential component required for heterochromatin assembly, but is an auxiliary factor that helps maintain integrity and repressive histone modifications at constitutive heterochromatin.

### Role of Set3 in heterochromatin silencing depends on PHD finger, but is independent of Set3C

**Figure 2:**
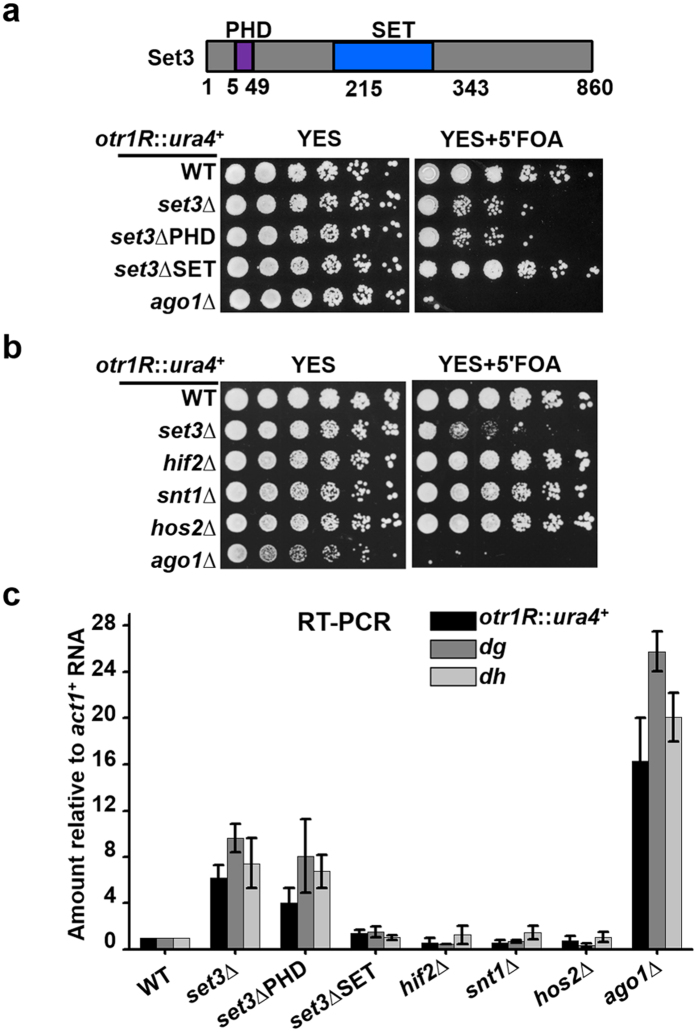
The role of Set3 in heterochromatin silencing relies on its PHD finger, but is independent of Set3C. (**a**) A schematic representation of domains in Set3 (top). A fivefold serial dilution assay to examine the silencing of *otr1R::ura4* in the mutants containing deletion of indicated domain (bottom). (**b**) A fivefold serial dilution assay to examine the silencing of *otr1R::ura4*^+^ in deletion mutants of Set3C subunits. (**c**) RT-PCR analysis of *otr1R::ura4*^+^, pericentromeric repeats (*dg* and *dh*) RNA levels relative to a control *act1*^+^. The relative level in WT cells was arbitrarily designated as 1. Each column represents the mean ± s.d. from three biological repeats.

Set3 harbors a PHD finger close to its N terminus, and a SET domain around middle region (Fig. 2a). To investigate the contribution of both domains to Set3-mediated silencing, *set3*ΔPHD and *set3*ΔSET mutants were constructed in the strain carrying an *otr1R::ura4*^+^ reporter. Deletion of either PHD or SET domain does not cause the degradation of protein (Supplementary Fig. S1). The growth of *set3*ΔPHD mutant on the 5′-FOA containing medium was as poor as in a *set3*Δ deletion mutant. In contrast, the *set3*ΔSET mutant grew as well on 5′FOA as WT (Fig. 2a). Consistently, the transcript levels of *otr1R::ura4*, *dg* and *dh* increased in *set3*ΔPHD, but not in a *set3*ΔSET mutant (Fig. 2c). The results indicate that the role of Set3 in heterochromatin silencing depends on its PHD finger, but not its SET domain.

In budding yeast, the PHD finger of Set3 recognizes H3K4me2 and is proposed to mediate the recruitment of Set3C to 5′ transcribed regions^26^. Therefore, we investigated whether other subunits of Set3C participate in the Set3-mediated silencing. Unexpectedly, in the strain bearing *otr1R::ura4*^+^, deletion of the catalytic subunit Hos2, or the structural subunits, including Snt1 and Hif2, did not affect the normal growth of the cells on 5′-FOA (Fig. 2b). Accordingly, transcript levels of *otr1R::ura4*^+^, *dg* and *dh* in *hos2*∆, *snt1*∆ and *hif2*∆ mutants were kept low as in WT cells (Fig. 2c). The results suggest that the pericentromeric region is silenced normally upon disruption of Set3C. Therefore, the role of Set3 in heterochromatin silencing is independent of enzymatic activity or the structural integrity of Set3C.

### Set3 promotes the transcription of *clr4* ^+^ and *rik1* ^+^

**Figure 3:**
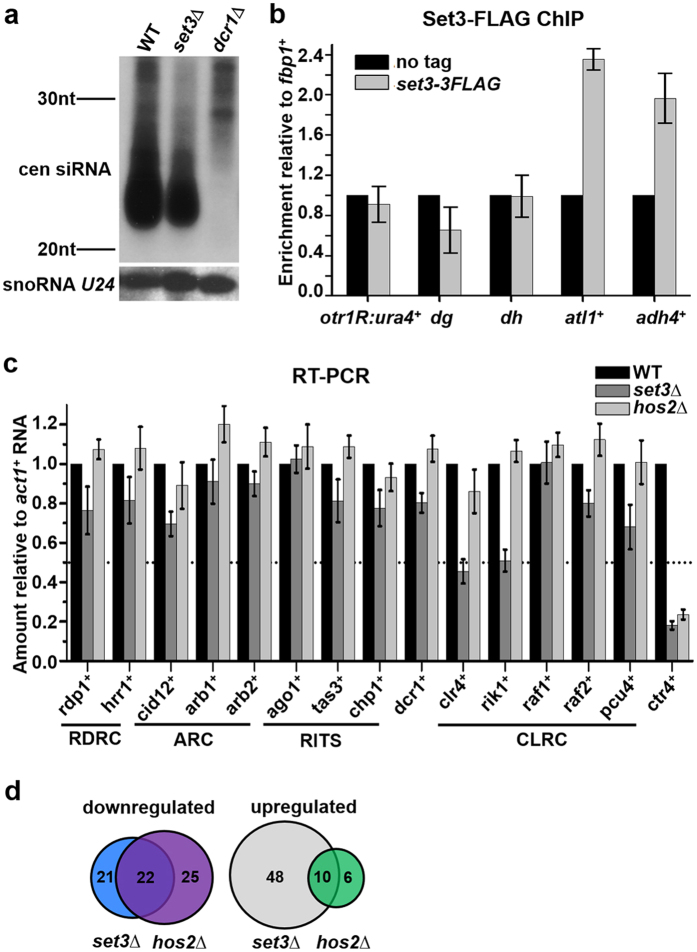
Set3 promotes the transcription of *clr4*^+^ and *rik1*^+^. (**a**) Northern blot analysis of centromeric siRNAs using probes against *dg* and *dh*. snoRNA *U24* was detected as a loading control. (**b**) ChIP analysis of enrichment of Set3-3FLAG at *otr1R::ura4*^+^, *dg*, *dh*, *atl1*^+^ and *adh4*^+^ relative to *fbp1*^+^. *fbp1*^+^ is not subjected to the regulation of Set3 or Hos2 (Supplementary Fig. S3). Relative enrichment in the cells with no tag was arbitrarily designated as 1. Each column shown in (**b**) and below represents the mean ± s.d. from three biological repeats. (**c**) RT-PCR analysis of RNA levels of essential components of RNAi machinery and CLRC in *set3*∆ and *hos2∆* mutants. The relative level to a control *act1*^+^ in WT cells was arbitrarily designated as 1. (**d**) Schematic representations of the genes downregulated or upregulated in the *set3*Δ and *hos2*Δ mutants, which were revealed by mRNA-seq.

Since Set3 acts independently of Set3C in heterochromatin silencing, this novel role Set3 might have evolved in fission yeast to cope with different heterochromatin effectors not present in *Saccharomyces cerevisiae*, such as RNAi and CLRC. To analyze the potential role of Set3 in RNAi, we performed a Northern blotting to detect siRNAs level in a *set3*∆ mutant. siRNAs corresponding to the pericentromeric repeats (*dg* and *dh*) were slightly decreased in a *set3*∆ mutant compared to WT, while the level of non-coding snoRNA *U24* was not affected (Fig. 3a). As a control, pericentromeric siRNAs were completely lost in *dcr1*∆ cells. The result is consistent with a weak phenotype of *set3*Δ mutant in heterochromatin silencing and suggests Set3 plays a non-essential role in the production of pericentromeric siRNAs.

Most of the RNAi components are localized at heterochromatin. To investigate whether Set3 is targeted to the pericentromeric region, we constructed a strain expressing Set3 with a C-terminal triple FLAG tag (Set3-3FLAG) and performed a ChIP assay. Note that this tag does not affect the gene silencing function of Set3 (Supplementary Fig. S2). Compared with an irrelevant euchromatic locus (*fbp1*^+^), Set-3FLAG was not enriched at the endogenous centromeric repeat (*dh*) and a inserted marker (*otr1R*::*ura4*^+^) (Fig. 3b). In contrast, substantial enrichment of Set3-3FLAG was detected at the ORFs of *atl1*^+^ and *adh4*^+^, both of which are known to be regulated by Set3^23^. Thus, Set3 does not regulate silencing by directly binding to the centromeric chromatin.

Next, we investigated whether Set3 is involved in the transcriptional regulation of factors required for heterochromatin formation. The mRNA levels of essential components of RNAi machinery and CLRC were measured in WT and *set3*Δ cells. These factors include Rdp1, Hrr1 and Cid12 from RDRC; Arb1 and Arb2 from ARC; Ago1, Tas3 and Chp1 from RITS; Dcr1; Clr4, Rik1, Raf1, Dos2 and Pcu4 from CLRC. As shown in Fig. 3c, mRNA levels of *clr4*^+^ and *rik1*^+^ were reduced substantially in a *set3*Δ mutant. In contrast, there are no significant changes of mRNA levels in a *hos2*Δ mutant. Since Hos2 is the catalytic subunit of Set3C, these results suggest Set3-mediated transcription of *clr4*^+^ and *rik1*^+^ is irrelevant of the activity of Set3C. This conclusion is consistent with a Set3C-independent role of Set3 in the heterochromatin silencing.

**Figure 4:**
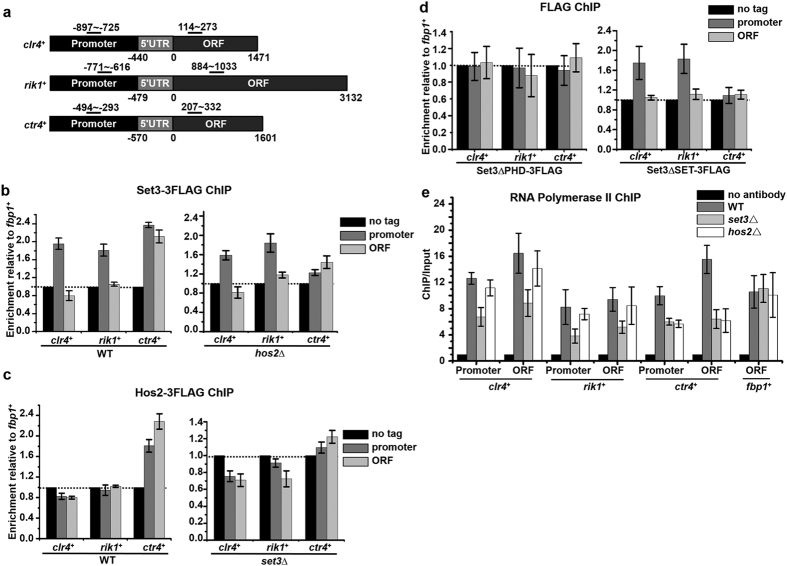
Set3 localizes to the promoters of *clr4*^+^ and *rik1*^+^ through its PHD finger. (**a**) Schematic representations of *clr4*^+^*, rik1*^+^ and *ctr4*^+^ loci. Black bars indicate the fragments amplified in ChIP assay. (**b–d**) ChIP analysis of enrichments of Set3-3FLAG (**b**), Hos2-3FLAG (**c**), Set3ΔPHD-3FLAG (**d**) and Set3ΔSET-3FLAG (**d**) at promoter and ORF region of *clr4*^+^*, rik1*^+^, or *ctr4*^+^ in the indicated strains. Relative enrichment to *fbp1*^+^ in the cells with no tag was arbitrarily designated as 1. Each column shown in (**b**) and below represents the mean ± s.d. from three biological repeats. (**e**) ChIP analysis of enrichments of RNA Pol II at promoter and ORF region of *clr4*^+^*, rik1*^+^*, ctr4*^+^ or *fbp1*^+^in the indicated strains. Relative enrichment in the no antibody control was arbitrarily designated as 1.

Noting the genes differentially regulated by Set3 and Hos2, we generated expression profiles for WT, *set3*∆ and *hos2*∆ cells by mRNA-seq. 22 genes were downregulated and 10 genes were upregulated in both *set3*Δ and *hos2*Δ mutants, suggesting that these genes are subjected to regulation by Set3C (Fig. 3d, Supplementary Table S1). Among the downregulated genes, *ctr4*^+^ encodes a copper transporter complex subunit^32^. The positive regulation of *ctr4*^+^ by Set3 and Hos2 was further verified by RT-PCR and ChIP (Figs 3c and 4b,c), and thus *ctr4*^+^ serves as a control of Set3C-targeted gene in the following study. Meanwhile, 48 genes were upregulated and 21 genes were downregulated in a *set3*Δ mutant independently of Hos2 (Fig. 3d, Supplementary Table S1). This suggests a Hos2-independent role of Set3 in transcription is not rare in fission yeast.

### Set3 localizes at the promoters of *clr4* ^+^ and *rik1* ^+^ through its PHD finger

To gain insight into the mechanism by which Set3 promotes the transcription of *clr4*^+^ and *rik1*^+^, the enrichment of Set3-3FLAG at both gene loci was investigated. As controls, the localizations of Set3 and Hos2 at *ctr4*^+^ locus was also determined (Fig. 4a). As shown in Fig. 4b, Set3 specifically localized at the promoter of *clr4*^+^ and *rik1*^+^, but not in their ORF region. There are several lines of evidence to support this localization is independent of Set3C. First, enrichment of Set3 at the promoters of *clr4*^+^ and *rik1*^+^ was not affected by the deletion of Hos2 (Fig. 4b). Second, Hos2 is not enriched at either the promoter or the ORF of either gene (Fig. 4c). Third, the conventional localization region of Set3C is not limited to the promoter region^26^. Consistently, Set3 and Hos2 were enriched at both the promoter and ORF region of *ctr4*^+^. The localization of Set3 at *ctr4*^+^ locus was abolished by the deletion of Hos2, and that of Hos2 was disrupted by the deletion of Set3, indicating *ctr4*^+^ is a *bona fide* target of Set3C (Fig. 4b,c). Therefore, our results suggest Set3 promotes the transcription of *clr4*^+^ and *rik1*^+^ by directly targeting the promoters of both genes, in a Set3C-independent manner.

The contributions of PHD finger and SET domain in the recruitment were investigated. Set3ΔPHD is not enriched at the promoters of *clr4*^+^ and *rik1*^+^, while Set3ΔSET is enriched at the promoters of both genes just like full-length Set3 (Fig. 4d). This suggests PHD finger, but not SET domain, is required for the localization of Set3 at the promoters of *clr4*^+^ and *rik1*^+^. Intriguingly, neither Set3ΔPHD nor Set3ΔSET is enriched at *ctr4*^+^ locus, suggesting PHD finger and SET domain are both required for the recruitment of Set3C to its target genes (Fig. 4d). The results strengthen the conclusion that Set3 adopts a Set3C-independent way to regulate the transcription of *clr4*^+^ and *rik1*^+^.

The role of Set3 in the transcription of *clr4*^+^ and *rik1*^+^ was further confirmed by a ChIP assay of RNA Pol II. As shown in Fig. 4e, occupancy of RNA Pol II at the promoters and coding regions of *clr4*^+^ and *rik1*^+^ was reduced substantially in *set3*Δ mutant, while no significant reduction was observed in *hos2*Δ mutant. This is consistent with the reduced mRNA levels of *clr4*^+^ and *rik1*^+^ in the *set3*Δ cells (Fig. 3C).

### Heterochromatin silencing defect of *set3*Δ mutant is suppressed by overexpressing *clr4* ^+^ or *rik1* ^+^

**Figure 5:**
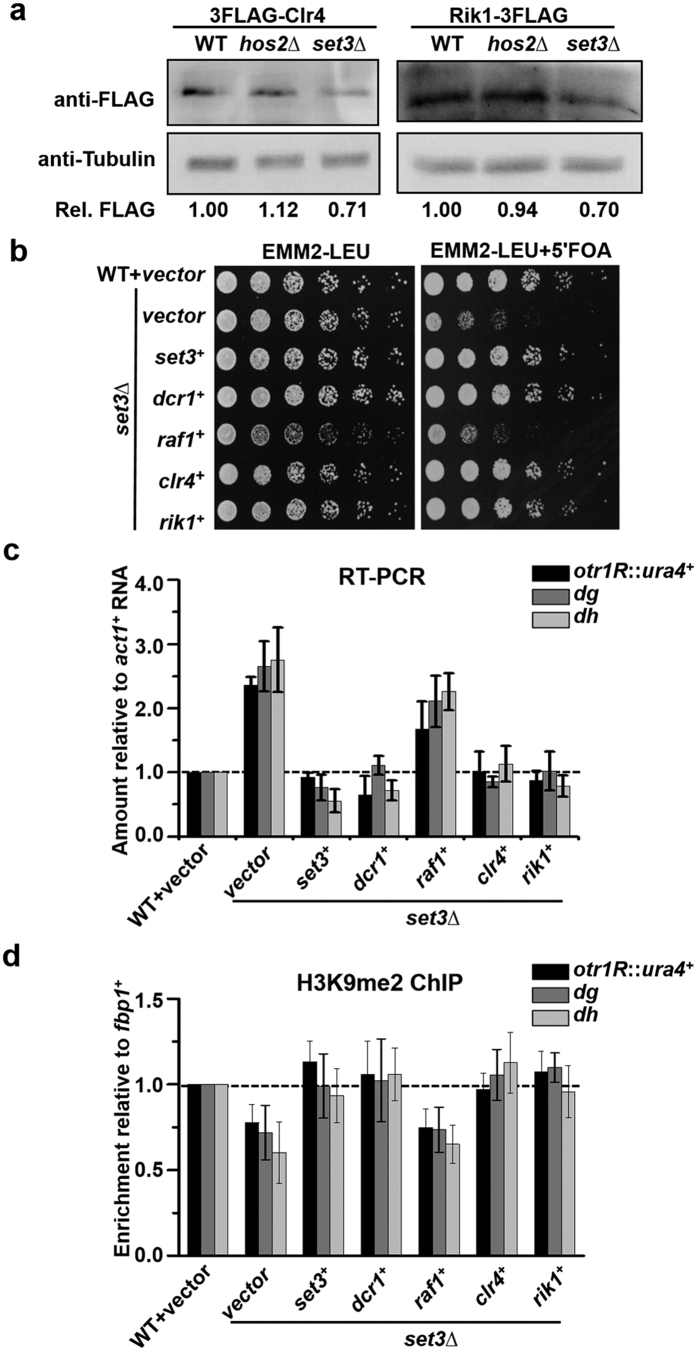
Heterochromatin silencing defect of *set3*Δ mutant is suppressed by overexpressing *clr4*^+^ or *rik1*^+^. (**a**) Western blotting assay to examine the level of 3FLAG-Clr4 and Rik1-3FLAG in WT, *set3*∆ and *hos2*∆ cells. Tubulin was detected as a loading control. Cropped blots are shown for clarity. Full-length blots are presented in Supplementary Fig. S5. The relative level of FLAG tagged protein (Rel. FLAG) was calculated by quantifying the relative density of 3FLAG-Clr4 or Rik1-3FLAG against tubulin. (**b**) Fivefold serial dilution assay to examine the silencing of *otr1R::ura4*^+^ in *set3*∆ cells overexpressing *set3*^+^, *dcr1*^+^, *raf1*^+^, *clr4*^+^ or *rik1*^+^. *set3*∆ cells were transformed with a plasmid overexpressing the indicated gene and transformants were subject to the dilution assay. (**c**) RT-PCR analysis of *otr1R::ura4*^+^, *dg* and *dh* RNA levels relative to a control *act1*^+^ in the transformants in (**b**). The relative level to a control *act1*^+^ in WT cells was arbitrarily designated as 1. Each column in (**c**) and below represents the mean ± s.d. from three biological repeats. (**d**) ChIP analysis of H3K9me2 at *otr1R::ura4*^+^, *dg* and *dh* relative to *fbp1*^+^ in the transformants in (**b**). Relative enrichment in WT cells was arbitrarily designated as 1.

As Set3 promotes the transcription of *clr4*^+^ and *rik1*^+^, we investigated whether the protein level of both factors is regulated by Set3 accordingly. To this end, Set3 was deleted in a strain expressing FLAG-tagged Rik1 or Clr4. The tags do not affect heterochromatin silencing (Supplementary Fig. S4). The protein levels of Clr4 and Rik1 in the *set3*Δ mutant reduced by 30% compared to those in WT cells. As expected, deletion of Hos2 had no apparent effect on the protein levels of both factors (Fig. 5a). H3K9 methylation declines rapidly upon the transient depletion of Clr4 or Rik1^19^,^33^. Thus, reduced protein levels of Clr4 and Rik1 are consistent with the decrease of H3K9me2 in *set3*Δ mutant (Fig. 1e,g).

To test whether the reduction of Clr4 and Rik1 accounts for the pericentromeric silencing defect of the *set3*Δ mutant, we performed a complementation assay. *set3*∆ mutant was transformed with a plasmid overexpressing *clr4*^+^, *rik1*^+^, *raf1*^+^, *set3*^+^, or *dcr1*^+^. The overexpression of genes was verified by RT-PCR (Supplementary Fig. S6). The silencing defect of *otr1R::ura4*^+^ in the *set3*∆ mutant was substantially rescued by overexpressing either *clr4*^+^ or *rik1*^+^, as shown by the improved growth on 5′FOA plate. In contrast, overexpressing *raf1*^+^, whose transcription is not affected by the deletion of Set3, could not rescue the silencing defect of the *set3*∆ mutant (Fig. 5b). Consistent with the good growth on 5-FOA plate, transcript levels of *ura4*^+^ and centromeric repeats (*dh*, *dg*) decreased in *set3*Δ cells overexpressing *clr4*^+^ or *rik1*^+^, but not in the cells overexpressing *raf1*^+^ (Fig. 5c). Dcr1 interacts with Clr4 and Rik1. Overexpressed Dcr1 promotes CLRC recruitment and H3K9 methylation at the centromeric region^34^. As shown in Fig. 5b, overexpressing *dcr1*^+^ was also able to rescue the growth defect of *set3*∆ cells on 5′-FOA, suggesting a connection between the Dcr1 and the Set3-regulated function of CLRC. Accordingly, transcript levels of *ura4*^+^ and *dh*/*dg* decreased in *set3*Δ mutant overexpressing *dcr1*^+^ (Fig. 5c). The integrity of pericentromeric heterochromatin was further investigated by a ChIP assay of H3K9me2. Levels of H3K9me2 at *otr1R*::*ura4*^+^, *dg* and *dh* in the *set3*Δ mutant were recovered by overexpressing *clr4*^+^, *rik1*^+^, *dcr1*^+^, but not by overexpressing *raf1*^+^ (Fig. 5d). These results suggest impaired silencing and integrity of heterochromatin in *set3*Δ mutant is mainly due to the reduced protein levels of Clr4/Rik1 and interfered H3K9 methylation by CLRC.

## Discussion

The interplay between the components essential for the heterochromatin formation and silencing is commonly mediated by physical interactions among the various proteins. But recently, the transcriptional regulation of heterochromatin factors emerges as a novel way to regulate heterochromatin assembly. Cwf14-mediated splicing of mRNA of RNAi factors, including Ago1, Arb2, Ers1, and Dsh1, is critical for pericentromeric heterochromatin assembly^35^. Tls1-mediated splicing of shelterin mRNAs regulates telomeric heterochromatin formation^36^. However, none of CLRC subunits contain intron, thus they are not subjected to the regulation by spliceosome. In this study, we reveal a novel role of Set3 in maintaining the integrity of heterochromatin by promoting the transcription of Clr4 and Rik1, two key components of CLRC. Decreased transcription resulted in the reduction of protein levels of both factors in the *set3*Δ mutant. Since the expression of Clr4 and Rik1 is not totally abolished, a limited effect on the function of CLRC, including assembly, targeting and the catalytic reactions, is expected in a *set3*Δ mutant. Accordingly, a relative mild effect on integrity and silencing of constitutive heterochromatin is observed in the *set3*Δ mutant (Fig. 1c–g). A weak effect on the production of pericentromeric siRNA is also observed (Fig. 3a). As H3K9me stabilizes the interaction between RITS and chromatin^10^, decreased H3K9me at pericentromeric region in the *set3*Δ mutant might affect the proper recruitment of RITS and following siRNA production. The weak phenotype of *set3*Δ mutant is contrast with the dramatic defects observed in the deletions of subunit of CLRC, or deletions of other factors that directly participate in the heterochromatin assembly^37^,^38^,^39^. Therefore, Set3 is not an on/off regulator, but is more like a fine-tuning factor contributing to the tight regulation of heterochromatin integrity at centromeres, telomeres and silent mating-type locus. Besides the constitutive heterochromatin region, low levels of CLRC binds with euchromatin sites, including noncoding RNAs, intergenic regions and meiotic genes^13^. Whether the disruption of CLRC by the deletion of Set3 imposes more significant effects on the its targets in the euchromatin region is worth studying in the future.

Our study indicates that Set3 is able to promote transcription in a Set3C-independent way. This novel role of Set3 might have evolved to target new genes absent in budding yeast, such as *clr4*^+^ and *rik1*^+^. Set3C is predominantly localized to the 5′ transcribed region and promotes transcription by inhibiting the cryptic transcription inside the ORF^26^. In fission yeast, targeting of Set3C seems require both PHD finger and SET domain of Set3. In contrast, Set3 is specifically recruited to the promoters of *clr4*^+^ and *rik1*^+^. The recruitment is probably mediated by the interaction between PHD finger and H3K4me2^26^, while SET domain is not necessary. Localization at the promoters suggests that Set3 contributes to the transcription initiation of *clr4*^+^ and *rik1*^+^, but underlying mechanism requires further investigation.

## Methods

### Yeast strains and plasmids

All the strains used in this study are listed in Supplementary Table S2. Gene deletion and tagging were performed by homologous recombination using a plasmid-based method^40^. Cells were grown in yeast extract medium with supplements (YES) or Edinburgh minimal medium minus leucine (EMM2-LEU) medium^41^. ORF of *set3*^+^, *rik1*^+^, *raf2*^+^, *clr4*^+^ or *dcr1*^+^ was cloned into pRep41 vector for overexpression^42^.

### Fivefold serial dilution assay

Exponentially growing cells were collected and adjusted to an A_600_ of 1.0. Samples were diluted by fivefold for five times. 5 μl dilutions were spotted onto YES or EMM2-LEU medium supplemented with 5′FOA (003234, Fluorochem, Hadfield, UK, 1 g/Liter) as indicated. Plates were incubated for 2 or 3d at 32 °C before imaging.

### RT-PCR

1 × 10^8^ exponentially growing cells were harvested. Total RNA were extracted using the RiboPure Yeast Kit (AM1926, Life Technologies, Carlsbad, CA, USA) and reverse transcribed into cDNA by using PrimeScript RT (RR037A, Takara, Dalian, China). qPCR was performed using SYBR Premix Ex TaqII (RR820A, Takara) in a LightCycler 480 II Real-Time PCR System (Roche Applied Science, Penzberg, Upper Bavaria, Germany). Primers used are listed in Supplementary Table S3.

### ChIP

3 × 10^8^ exponentially growing cells were fixed with 1% formaldehyde for 25 min at 30 °C. After quenching by 250 mM glycine, cells were harvested and washed with Buffer 1 (50 mM HEPES (pH 7.5), 140 mM NaCl, 1 mM EDTA, 1% TritonX-100, 0.1% Na-deoxycholate). Cells were resuspended in Buffer 1 supplemented with protease inhibitors cocktail (05892970001, Roche Applied Science) and homogenized with a bead-beater (FastPrep-24, MP, California, USA) by glass beads. The cell extract was sonicated for 8 min at high frequency with a sonication system (Bioruptor UCD-300, Diagenode, Seraing, Belgium) and centrifuged. Supernatant was incubated with anti-H3K9me2 (07-441, Millipore, Massachusetts, USA), anti-RNA polymerase II 8WG16 (664906, Biolegend, San Diego, USA), or anti-FLAG (F1804, Sigma-Aldrich, St Louis, MO, USA) antibody for 4 hours. Samples were subjected to purification by using an EZ-Magna ChIP A Kit (17-408, Millipore). Eluted DNA was subjected to qPCR as described above. Primers used are listed in Supplementary Table S3.

### Northern blot

Northern blot was performed as described previously^39^. Primers and oligos used are listed in Supplementary Table S3.

### Whole cell extract

Exponentially growing yeast cells were harvested. Cells were washed and resuspended in ice-cold Buffer 1 supplemented with protease inhibitors as described above. Cells were broken in a bead-beater by glass beads. Extract was centrifuged at 4 °C. Supernatant was boiled with SDS-gel loading buffer and subjected to Western blotting by using anti-FLAG and anti-Tubulin (T6074, Sigma) antibodies. The blots were scanned and quantified by GeneGnome HR system (Syngene, Cambridge, UK).

### mRNA-seq

Exponentially growing yeast cells were harvested and total RNA was extracted by using RiboPure Yeast Kit. Enrichment of mRNA, fragmentation, reverse transcription, library construction, Hi-seq and data analysis were performed by Genergy Biotechnology (Shanghai, China).

## Additional Information

**How to cite this article**: Yu, Y. *et al*. Set3 contributes to heterochromatin integrity by promoting transcription of subunits of Clr4-Rik1-Cul4 histone methyltransferase complex in fission yeast. *Sci. Rep.* **6**, 31752; doi: 10.1038/srep31752 (2016).

## Supplementary Material

Supplementary Information
