# Supplementary material for: Set3 contributes to heterochromatin integrity by promoting transcription of subunits of Clr4-Rik1-Cul4 histone methyltransferase complex in fission yeast
Source: Sci Rep. 2016 Aug 19;6:31752. doi: 10.1038/srep31752 (PMC4990937; doi:10.1038/srep31752)
Supplement: Supplementary Information [file srep31752-s1.doc]

**Supplementary Information**

**Set3 contributes to heterochromatin integrity by promoting transcription of subunits of Clr4-Rik1-Cul4 histone methyltransferase complex in fission yeast**

Yao Yu^1,2#^*, Huan Zhou^1,2#^, Xiaolong Deng^1,2^, Wenchao Wang^1,2^, Hong Lu^1,2,3^

^1^ State Key Laboratory of Genetic Engineering, School of Life Sciences, Fudan University

^2^ Shanghai Engineering Research Center Of Industrial Microorganisms, Shanghai, China, 200438

^3^ Shanghai Collaborative Innovation Center for Biomanufacturing Technology, Shanghai, China, 200237

Correspondence and requests for materials should be addressed to Y.Y. (email: [yaoyu@fudan.edu.cn](mailto:yaoyu@fudan.edu.cn))

#These authors contributed equally to this work.

**
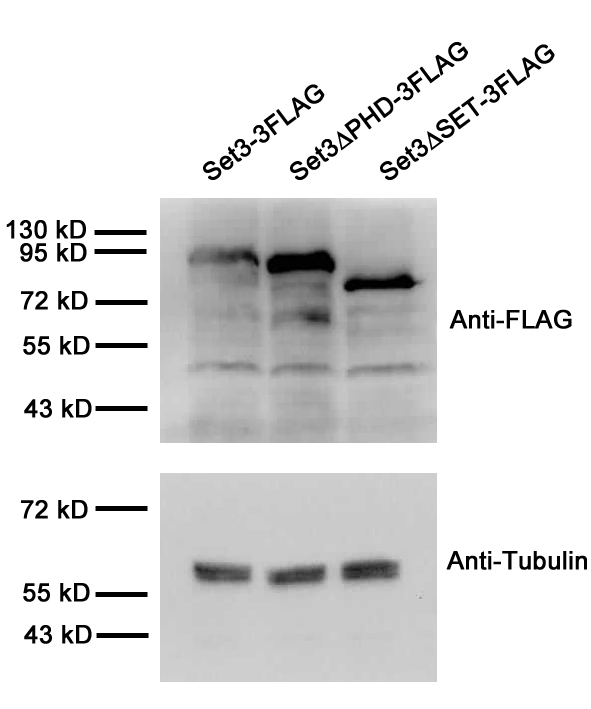
**

**Supplementary Figure S1. Expression of Set3-3FLAG, Set3ΔPHD-3FLAG and Set3ΔSET-3FLAG.**

Western blotting assay to examine the levels of Set3-3FLAG, Set3ΔPHD-3FLAG and Set3ΔSET-3FLAG. Tubulin was detected as a loading control.


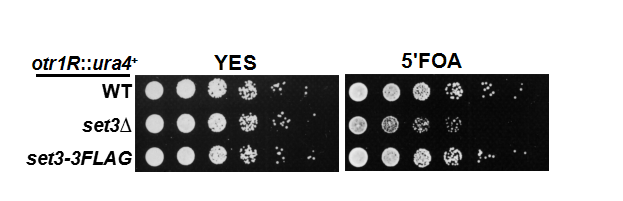


**Supplementary Figure S2. Set3-3FLAG strain displayed normal silencing at pericentromeric region.**

Fivefold serial dilution assay to examine the silencing of *otr1R*::*ura4*^+^ in strain expressing Set3 with a C terminal triple FLAG tag (Set3-3FLAG). Wild-type (WT) cells with no tag and a *set3*Δ mutant were assayed as controls.


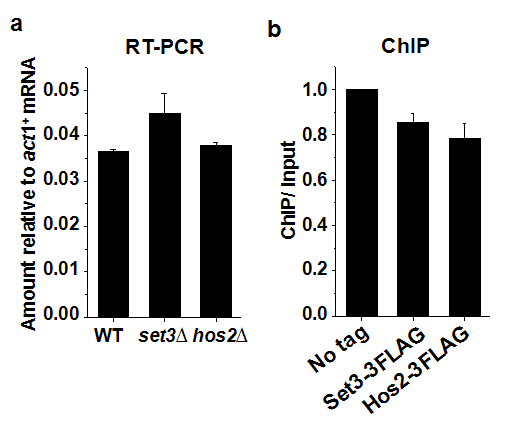


**Supplementary Figure S3. *fbp1*^+^ is not regulated by Set3 or Hos2**

**(a)** RT-PCR analysis of RNA levels of *fbp1*^+^ in WT, *set3*∆ and *hos2*∆ relative to a control *act1*^+^. Each column shown in (a) and below represents the mean ± s.d. from three biological repeats. **(b)** ChIP analysis of enrichment of Set3-3FLAG and Hos2-3FLAG at *fbp1*^+^. Relative enrichment in the cells with no tag was arbitrarily designated as 1.

**
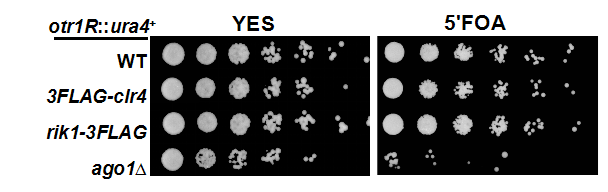
**

**Supplementary Figure S4. 3FLAG-Clr4 and Rik1-3FLAG strain displayed normal silencing at pericentromeric region.**

Fivefold serial dilution assay to examine the silencing of *otr1R*::*ura4*^+^ in strain expressing Clr4 with a N terminal triple FLAG tag (3FLAG-Clr4) or Rik1 with a C terminal triple FLAG tag (Rik1-3FLAG).


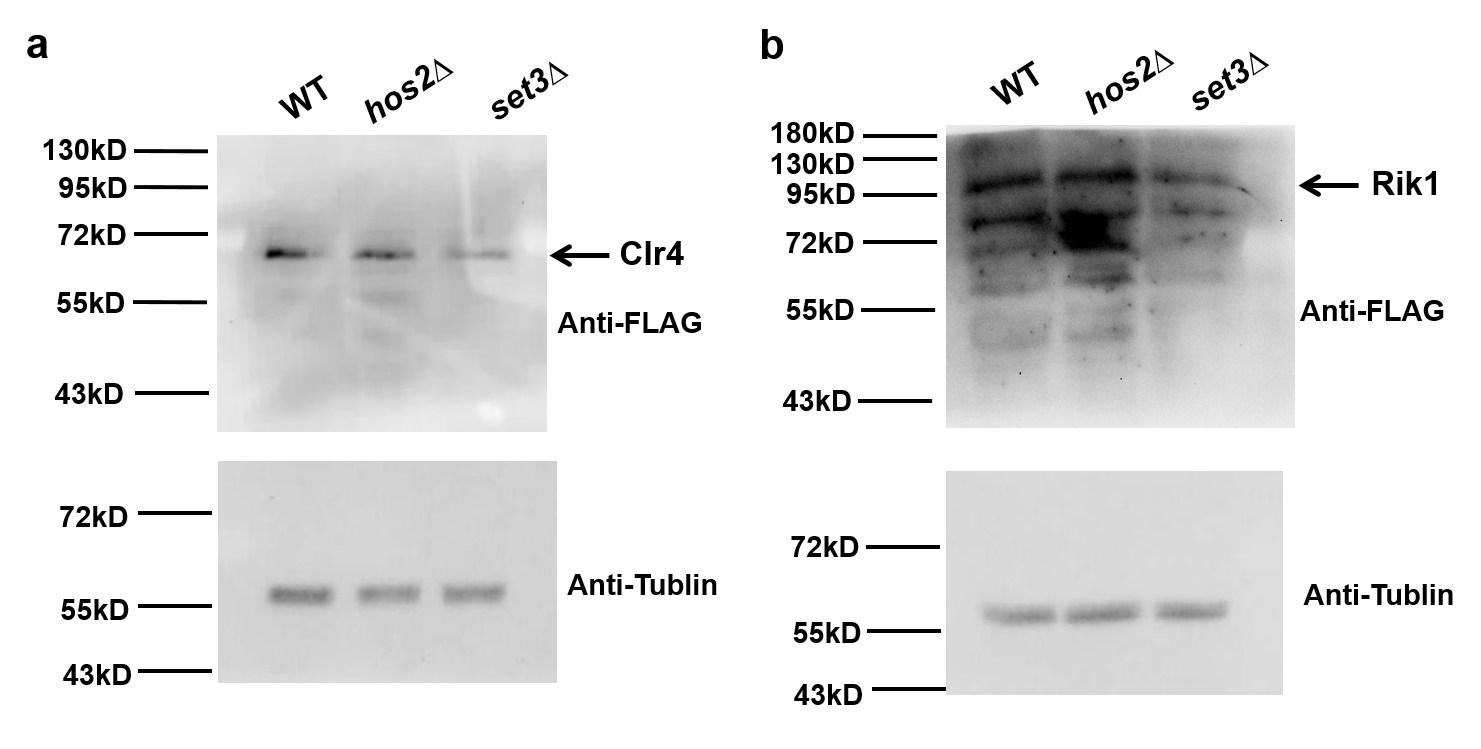


**Supplementary Figure S5. Full-length blots in Fig. 5a**

1. The Western blotting of 3FLAG-Clr4 and tubulin in whole cell extract from WT, *set3*∆ and *hos2*∆ cells. The MW of 3FLAG-Clr4 is about 60 kDa.
2. The Western blotting of Rik1-3FLAG and tubulin in whole cell extract from WT, *set3*∆ and *hos2*∆ cells. The MW of Rik1-3FLAG is about 120kDa.


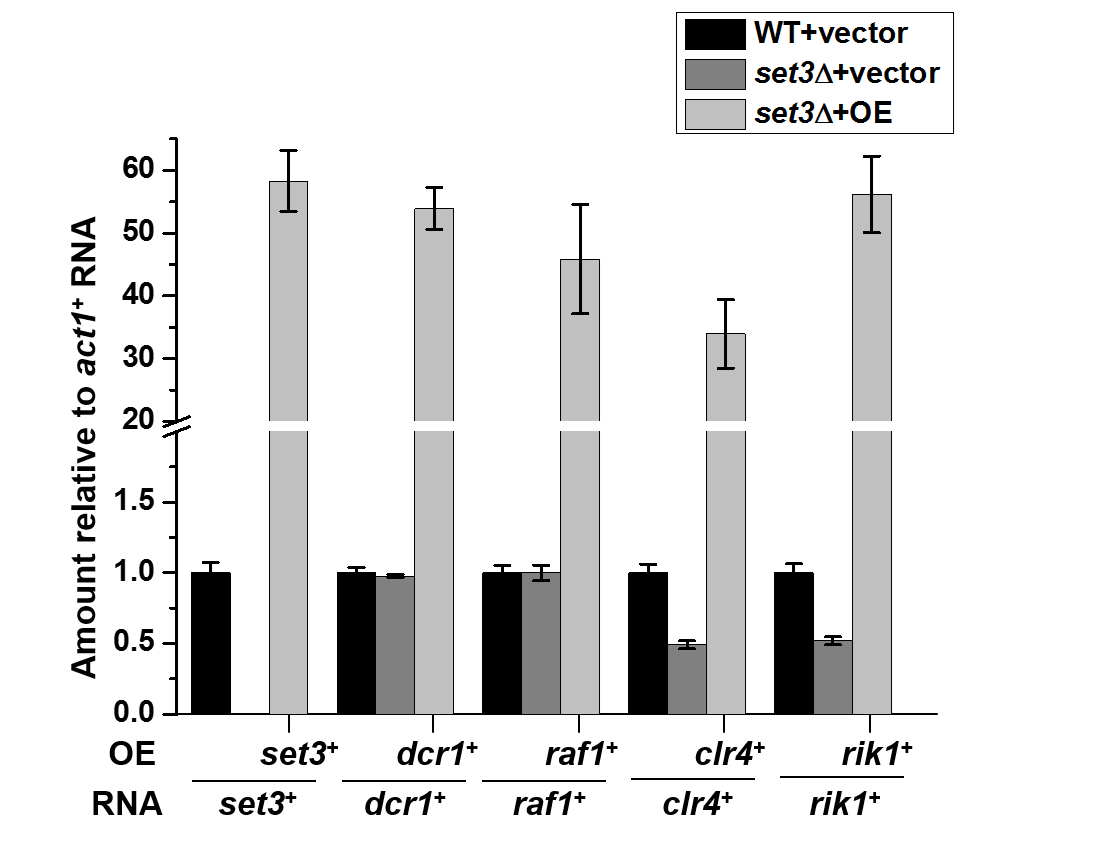


**Supplementary Figure S6. Overexpression of *set3*^+^, *dcr1*^+^, *raf1*^+^, *clr4*^+^ and *rik1*^+^ in the transformants.**

*set3*Δ cells were transformed with pRep41 plasmids overexpressing (OE) indicated genes. mRNA levels of indicated genes in the transformants were analyzed by RT-PCR. The relative level in WT cells transformed with pRep41 vector was arbitrarily designated as 1. Each column represents the mean ± s.d. from three biological repeats.

**Supplementary Table S1. List of genes whose mRNA levels were affected by the deletion of *set3*^+^ or *hos2*^+^ in mRNA seq.**

**Table S1-1. Downregulated genes in *set3*∆ mutant**

| **gene** | **value_WT** | **value_set3∆** | **log2(fold_change)** | ***p*_value** | ***q*_value** |
| --- | --- | --- | --- | --- | --- |
| **SPAC869.08** | **7.33228** | **0.438357** | **-4.06408** | **0.0491** | **0.99915** |
| SPAC212.07c | 2.01321 | 0.148063 | -3.76521 | 0.01075 | 0.843535 |
| SPNCRNA.1326 | 4.29526 | 0.390576 | -3.45907 | 0.0155 | 0.99915 |
| **SPNCRNA.774** | **26.7499** | **2.88218** | **-3.2143** | **0.00985** | **0.803423** |
| **SPAC29A4.12c** | **55.7647** | **9.52343** | **-2.5498** | **0.00295** | **0.331701** |
| SPNCRNA.1299 | 43.4417 | 7.55419 | -2.52373 | 0.00345 | 0.362484 |
| SPBC1289.17 | 1.79795 | 0.320701 | -2.48706 | 0.03325 | 0.99915 |
| **SPCC645.14c** | **495.209** | **121.472** | **-2.02742** | **5.00E-05** | **0.0140886** |
| **SPCC1393.10**  ***ctr4*^+^** | **578.346** | **143.712** | **-2.00876** | **5.00E-05** | **0.0140886** |
| **SPNCRNA.1036** | **641.064** | **159.984** | **-2.00254** | **0.00015** | **0.0344389** |
| **SPAC186.01** | **6.95806** | **1.80578** | **-1.94606** | **0.04245** | **0.99915** |
| SPNCRNA.1190 | 16.0563 | 4.20732 | -1.93217 | 0.0186 | 0.99915 |
| **SPAC13F5.03c** | **50.6879** | **14.3145** | **-1.82416** | **0.01075** | **0.843535** |
| SPNCRNA.1278 | 8.17831 | 2.31723 | -1.8194 | 0.02535 | 0.99915 |
| SPAC13G7.02c | 213.284 | 61.2085 | -1.80098 | 5.00E-05 | 0.0140886 |
| SPAC27D7.09c | 111.524 | 32.2153 | -1.79153 | 0.0001 | 0.0258292 |
| **SPBC3B9.01** | **268.644** | **78.6925** | **-1.7714** | **5.00E-05** | **0.0140886** |
| SPBC3E7.02c | 507.251 | 149.315 | -1.76434 | 5.00E-05 | 0.0140886 |
| **SPNCRNA.897** | **34.4077** | **10.6276** | **-1.69491** | **0.00355** | **0.366774** |
| **SPCC1739.13** | 3244.29 | 1024.87 | -1.66246 | 5.00E-05 | 0.0140886 |
| SPBC30D10.14 | 230.654 | 72.8763 | -1.66221 | 0.0001 | 0.0258292 |
| SPNCRNA.103 | 24.3021 | 7.91904 | -1.61769 | 0.0466 | 0.99915 |
| **SPAC926.04c** | **2074.02** | **701.895** | **-1.5631** | **0.00015** | **0.0344389** |
| SPAC513.02 | 23.6074 | 8.31585 | -1.50531 | 0.03075 | 0.99915 |
| SPAC9E9.11 | 514.055 | 185.199 | -1.47285 | 0.00015 | 0.0344389 |
| SPNCRNA.832 | 204.361 | 74.1224 | -1.46314 | 0.00825 | 0.720306 |
| **SPBC1711.08** | **395.364** | **143.552** | **-1.46161** | **0.00035** | **0.0699887** |
| **SPBPB2B2.06c** | **11.5775** | **4.39724** | **-1.39666** | **0.04575** | **0.99915** |
| **SPAC227.13c** | **217.784** | **84.2902** | **-1.36946** | **0.02105** | **0.99915** |
| **SPAC1006.08** | **41.8423** | **16.3327** | **-1.3572** | **0.0104** | **0.837268** |
| **SPNCRNA.1447** | **58.1401** | **23.1605** | **-1.32787** | **0.0016** | **0.241912** |
| **SPAC644.05c** | **239.474** | **96.4028** | **-1.31272** | **0.01685** | **0.99915** |
| **SPBC428.18** | **44.0717** | **18.1895** | **-1.27675** | **0.01215** | **0.896641** |
| SPAC29A4.11 | 9.16781 | 3.91587 | -1.22724 | 0.02415 | 0.99915 |
| **SPAC1250.04c** | **53.9778** | **23.453** | **-1.20259** | **0.00075** | **0.122349** |
| **SPNCRNA.923** | **421.577** | **187.942** | **-1.16551** | **0.0476** | **0.99915** |
| SPNCRNA.1397 | 10.7624 | 4.89988 | -1.13518 | 0.0402 | 0.99915 |
| SPNCRNA.1100 | 64.4169 | 29.7162 | -1.11619 | 0.01115 | 0.863986 |
| SPBC14F5.04c | 5556.71 | 2572.91 | -1.11083 | 0.0051 | 0.498826 |
| **SPAC1142.05** | **306.544** | **147.335** | **-1.057** | **0.02285** | **0.99915** |
| SPAC27D7.11c | 212.858 | 102.781 | -1.05033 | 0.00235 | 0.28564 |
| **SPBC660.14** | **24.0299** | **11.8339** | **-1.0219** | **0.0282** | **0.99915** |
| SPAC1F12.10c | 117.103 | 57.9289 | -1.01542 | 0.02155 | 0.99915 |

**Table. S1-2. Upregulated genes in *set3*∆ mutant.**

| **gene** | **value_WT** | **value_set3∆** | **log2(fold_change)** | ***p*_value** | ***q*_value** |
| --- | --- | --- | --- | --- | --- |
| **SPCC16C4.13c** | **688.804** | **1416.68** | **1.04035** | **0.0063** | **0.574319** |
| SPAC17A2.09c | 38.0279 | 79.329 | 1.06079 | 0.003 | 0.331701 |
| **SPBC32C12.02** | **11.7962** | **24.9768** | **1.08226** | **0.0084** | **0.723217** |
| SPAC1002.18 | 103.807 | 220.11 | 1.08432 | 0.0026 | 0.30995 |
| SPAPB1A10.12c | 90.6863 | 193.199 | 1.09113 | 0.00585 | 0.541256 |
| SPCC1235.14 | 489.012 | 1052.97 | 1.10653 | 0.00685 | 0.615408 |
| **SPCC4G3.10c** | **12.0136** | **25.9135** | **1.10903** | **0.01265** | **0.922557** |
| **SPBC1861.01c** | **53.6928** | **117.39** | **1.12851** | **0.00905** | **0.768506** |
| SPBP35G2.16c | 67.5483 | 148.771 | 1.1391 | 0.002 | 0.266557 |
| SPAC4A8.04 | 51.8419 | 114.634 | 1.14485 | 0.0349 | 0.99915 |
| SPBC3H7.02 | 46.5074 | 103.639 | 1.15603 | 0.0423 | 0.99915 |
| SPCC1183.11 | 14.8485 | 33.3739 | 1.1684 | 0.00335 | 0.358046 |
| SPAP7G5.06 | 10.637 | 23.9541 | 1.17118 | 0.03145 | 0.99915 |
| SPCC965.06 | 52.1585 | 123.452 | 1.24297 | 0.0007 | 0.117278 |
| SPBPB7E8.02 | 22.5975 | 53.9896 | 1.25652 | 0.0007 | 0.117278 |
| SPAC23H4.06 | 82.2128 | 198.927 | 1.27481 | 0.0007 | 0.117278 |
| SPBC32H8.02c | 7.35708 | 17.9137 | 1.28386 | 0.0114 | 0.872452 |
| SPBC2G5.06c | 383.203 | 939.529 | 1.29383 | 0.00195 | 0.266557 |
| **SPBC1683.01** | **46.9947** | **115.586** | **1.2984** | **0.00045** | **0.0845318** |
| SPAC110.01 | 8.97114 | 22.3692 | 1.31815 | 0.01215 | 0.896641 |
| SPAC23A1.14c | 64.4 | 165.547 | 1.36211 | 0.00025 | 0.0516583 |
| SPAC16E8.16 | 16.5404 | 42.6014 | 1.36491 | 0.00295 | 0.331701 |
| SPMTR.01 | 59.9565 | 155.402 | 1.37401 | 0.03125 | 0.99915 |
| **SPBP8B7.15c** | **12.7668** | **33.8691** | **1.40758** | **0.0029** | **0.331701** |
| **SPCC16C4.07** | **19.4516** | **51.8833** | **1.41538** | **0.00305** | **0.331701** |
| SPCC70.12c | 13.3353 | 35.6308 | 1.41788 | 0.01375 | 0.991119 |
| **SPAC4H3.08** | **13.182** | **38.3671** | **1.5413** | **0.00515** | **0.498826** |
| SPCC584.01c | 63.5767 | 197.403 | 1.63457 | 5.00E-05 | 0.0140886 |
| SPAC10F6.01c | 103.17 | 324.214 | 1.65192 | 5.00E-05 | 0.0140886 |
| SPCC31H12.02c | 7.99062 | 25.9233 | 1.69787 | 0.02215 | 0.99915 |
| SPNCRNA.1374 | 13.7136 | 44.533 | 1.69926 | 5.00E-05 | 0.0140886 |
| SPBPB2B2.01 | 6.68038 | 22.0239 | 1.72107 | 0.00525 | 0.500688 |
| SPBPB10D8.01 | 31.3761 | 104.551 | 1.73648 | 0.0038 | 0.386167 |
| SPNCRNA.388 | 3.96395 | 13.772 | 1.79672 | 0.0294 | 0.99915 |
| SPNCRNA.1696 | 39.9474 | 139.794 | 1.80713 | 0.0006 | 0.109394 |
| SPNCRNA.727 | 13.2616 | 50.0934 | 1.91737 | 0.0329 | 0.99915 |
| SPBC106.17c | 100.5 | 382.282 | 1.92745 | 0.00045 | 0.0845318 |
| SPNCRNA.1307 | 47.2095 | 186.306 | 1.98053 | 0.00025 | 0.0516583 |
| SPAC29B12.10c | 49.5485 | 202.7 | 2.03243 | 5.00E-05 | 0.0140886 |
| **SPAC27D7.03c** | **4.02596** | **16.6739** | **2.05019** | **0.00105** | **0.162724** |
| SPBC428.11 | 161.635 | 713.126 | 2.14141 | 5.00E-05 | 0.0140886 |
| SPAC57A10.05c | 19.3679 | 86.4432 | 2.15809 | 5.00E-05 | 0.0140886 |
| SPCC1739.06c | 86.5159 | 394.302 | 2.18826 | 5.00E-05 | 0.0140886 |
| SPBP16F5.08c | 90.0599 | 419.134 | 2.21846 | 5.00E-05 | 0.0140886 |
| SPBCPT2R1.07c | 0.909421 | 4.4903 | 2.30379 | 0.0421 | 0.99915 |
| **SPNCRNA.130** | **6.2442** | **33.9368** | **2.44226** | **0.00535** | **0.502495** |
| SPCPB1C11.01 | 9.93867 | 54.2784 | 2.44925 | 5.00E-05 | 0.0140886 |
| SPAC1002.17c | 136.189 | 759.205 | 2.47888 | 5.00E-05 | 0.0140886 |
| SPAC25B8.13c | 18.6296 | 103.993 | 2.48082 | 5.00E-05 | 0.0140886 |
| SPCPB1C11.03 | 14.3698 | 87.2237 | 2.60169 | 5.00E-05 | 0.0140886 |
| SPAC1399.04c | 43.8618 | 295.124 | 2.75028 | 5.00E-05 | 0.0140886 |
| SPBCPT2R1.08c | 0.408652 | 2.75936 | 2.75539 | 0.00755 | 0.668606 |
| SPAC212.11 | 0.208136 | 1.50613 | 2.85525 | 0.0211 | 0.99915 |
| SPAC869.05c | 52.3351 | 379.833 | 2.85951 | 5.00E-05 | 0.0140886 |
| SPAC1002.19 | 89.2243 | 657.861 | 2.88227 | 5.00E-05 | 0.0140886 |
| SPAPJ695.02 | 1.91276 | 15.2854 | 2.99843 | 0.00105 | 0.162724 |
| SPBC839.06 | 0.47474 | 4.52859 | 3.25385 | 0.04975 | 0.99915 |
| SPBPB2B2.08 | 5.81631 | 186.711 | 5.00456 | 5.00E-05 | 0.0140886 |
| dh | 134 | 832 | 2.634351 | - | - |
| dg | 132 | 420 | 1.669851 | - | - |
| otr1::ura4 | 38 | 188 | 2.306661 | - | - |

**Table. S1-3. Downregulated genes in *hos2*∆ mutant.**

| **gene** | **value_WT** | **value_hos2∆** | **log2(fold_change)** | ***p*_value** | ***q*_value** |
| --- | --- | --- | --- | --- | --- |
| SPAC3G9.07c | 37.7447 | 0.627642 | -5.91019 | 0.0033 | 0.731893 |
| **SPAC869.08** | **7.38487** | **0.529619** | **-3.80155** | **0.04475** | **0.99945** |
| **SPNCRNA.774** | **26.9418** | **4.33591** | **-2.63544** | **0.0122** | **0.99945** |
| SPNCRNA.287 | 26.0041 | 4.31233 | -2.5922 | 0.01535 | 0.99945 |
| SPNCRNA.650 | 7.74889 | 1.38584 | -2.48323 | 0.01815 | 0.99945 |
| SPNCRNA.448 | 4.49599 | 0.846116 | -2.40971 | 0.04315 | 0.99945 |
| **SPAC186.01** | **7.00797** | **1.3707** | **-2.35409** | **0.0461** | **0.99945** |
| SPNCRNA.362 | 6.37666 | 1.29713 | -2.29748 | 0.02195 | 0.99945 |
| **SPCC1393.10**  *ctr4*^+^ | 582.494 | 139.487 | -2.06211 | 5.00E-05 | 0.077625 |
| SPAC977.01 | 9.98267 | 2.41346 | -2.04833 | 0.04075 | 0.99945 |
| SPMTR.01 | 60.3865 | 15.7201 | -1.94162 | 0.0271 | 0.99945 |
| SPCC191.11 | 120.586 | 31.4828 | -1.93742 | 5.00E-05 | 0.077625 |
| SPBPB21E7.07 | 84.3731 | 22.1503 | -1.92946 | 0.0003 | 0.207 |
| **SPAC29A4.12c** | **56.1646** | **15.2317** | **-1.88258** | **0.0041** | **0.771545** |
| **SPBPB2B2.06c** | **11.6606** | **3.781** | **-1.6248** | **0.01695** | **0.99945** |
| SPBPB2B2.05 | 31.5831 | 10.392 | -1.60368 | 0.01225 | 0.99945 |
| **SPAC13F5.03c** | **51.0514** | **16.867** | **-1.59775** | **0.0072** | **0.99945** |
| SPBC1348.02 | 25.5366 | 8.75691 | -1.54407 | 0.0079 | 0.99945 |
| SPBPB21E7.09 | 13.4032 | 4.62159 | -1.53611 | 0.01265 | 0.99945 |
| SPNCRNA.953 | 73.1712 | 25.5811 | -1.5162 | 0.04365 | 0.99945 |
| **SPNCRNA.1036** | **645.662** | **229.527** | **-1.49212** | **0.0002** | **0.177429** |
| **SPBC3B9.01** | **270.57** | **102.095** | **-1.4061** | **0.0002** | **0.177429** |
| SPBC359.04c | 6.88024 | 2.62673 | -1.38919 | 0.04985 | 0.99945 |
| SPNCRNA.1305 | 58.9281 | 22.7626 | -1.37229 | 0.00135 | 0.46575 |
| **SPCC645.14c** | **498.761** | **193.954** | **-1.36263** | **5.00E-05** | **0.077625** |
| SPBPB21E7.04c | 66.8003 | 26.1674 | -1.35208 | 0.00455 | 0.816171 |
| **SPAC1142.05** | **308.743** | **121.047** | **-1.35084** | **0.0007** | **0.334385** |
| **SPNCRNA.923** | **424.601** | **167.595** | **-1.34113** | **0.0093** | **0.99945** |
| **SPBC428.18** | **44.3878** | **17.7908** | **-1.31903** | **0.0066** | **0.99945** |
| SPBC359.05 | 13.3259 | 5.43415 | -1.29411 | 0.0173 | 0.99945 |
| **SPAC1006.08** | **42.1424** | **17.561** | **-1.2629** | **0.0078** | **0.99945** |
| **SPAC644.05c** | **241.191** | **101.446** | **-1.24947** | **0.01265** | **0.99945** |
| SPNCRNA.1312 | 27.4179 | 11.6341 | -1.23676 | 0.02395 | 0.99945 |
| SPNCRNA.1302 | 23.3934 | 9.94911 | -1.23346 | 0.0389 | 0.99945 |
| **SPAC926.04c** | **2088.89** | **903.669** | **-1.20887** | **0.0004** | **0.2484** |
| **SPCC1739.13** | **3267.56** | **1415.97** | **-1.20643** | **0.00085** | **0.3519** |
| SPBC1683.09c | 69.8064 | 30.7303 | -1.1837 | 0.0006 | 0.334385 |
| **SPBC660.14** | **24.2023** | **11.0601** | **-1.12978** | **0.0111** | **0.99945** |
| **SPBC1711.08** | **398.199** | **182.469** | **-1.12584** | **0.001** | **0.388125** |
| **SPNCRNA.897** | **34.6544** | **16.0458** | **-1.11084** | **0.01205** | **0.99945** |
| SPCC1020.03 | 28.6048 | 13.6267 | -1.06982 | 0.0311 | 0.99945 |
| SPBC4F6.09 | 181.19 | 86.6296 | -1.06457 | 0.00115 | 0.420088 |
| SPAC23C4.13 | 126.489 | 60.6371 | -1.06074 | 0.00505 | 0.871125 |
| SPAC1F8.03c | 28.4232 | 14.0409 | -1.01744 | 0.0189 | 0.99945 |
| SPAC3G6.05 | 119.431 | 59.4138 | -1.0073 | 0.00605 | 0.988697 |
| **SPNCRNA.1447** | **58.5571** | **29.1378** | **-1.00695** | **0.00765** | **0.99945** |
| **SPAC227.13c** | **219.346** | **109.37** | **-1.00399** | **0.0371** | **0.99945** |

**Table. S1-4. Upregulated genes in *hos2*∆ mutant.**

| **gene** | **value_WT** | **value_hos2∆** | **log2(fold_change)** | ***p*_value** | ***q*_value** |
| --- | --- | --- | --- | --- | --- |
| SPAC20G8.09c | 59.6235 | 120.669 | 1.0171 | 0.00245 | 0.621 |
| SPBC1D7.03 | 48.5707 | 98.9065 | 1.02598 | 0.0021 | 0.592773 |
| SPRRNA.46 | 22.1254 | 45.914 | 1.05323 | 0.0099 | 0.99945 |
| **SPCC16C4.07** | **19.5911** | **40.7193** | **1.05551** | **0.01675** | **0.99945** |
| SPNCRNA.865 | 69.9432 | 147.626 | 1.07769 | 0.00225 | 0.6075 |
| **SPCC16C4.13c** | **693.745** | **1470.83** | **1.08415** | **0.00085** | **0.3519** |
| **SPCC4G3.10c** | **12.0998** | **26.0497** | **1.10628** | **0.00765** | **0.99945** |
| **SPBP8B7.15c** | **12.8583** | **27.8619** | **1.11559** | **0.0085** | **0.99945** |
| **SPBC1683.01** | **47.3318** | **103.127** | **1.12354** | **0.00065** | **0.334385** |
| SPAC2H10.01 | 43.0899 | 98.1399 | 1.18749 | 0.00025 | 0.194062 |
| **SPBC1861.01c** | **54.0779** | **127.845** | **1.24128** | **0.00155** | **0.506605** |
| SPBP8B7.16c | 178.223 | 450.506 | 1.33787 | 5.00E-05 | 0.077625 |
| **SPBC32C12.02** | **11.8808** | **30.8916** | **1.37858** | **0.00015** | **0.177429** |
| **SPAC4H3.08** | **13.2766** | **36.1365** | **1.44457** | **0.0052** | **0.872757** |
| **SPAC27D7.03c** | **4.05483** | **12.6657** | **1.64321** | **0.004** | **0.771545** |
| **SPNCRNA.130** | **6.28899** | **20.689** | **1.71796** | **0.0265** | **0.99945** |

mRNA seq was performed and analyzed by Genergy company (Shanghai, China). Differentiated regulated genes were listed, whose transcripts in mutant exhibited a change bigger 2 fold compared to WT cells while *p* value is smaller than 0.05. *q* value stands for the *p* value corrected by Benjamini-Hochberg correction methods. Genes up-regulated or down-regulated in both *set3*∆ and *hos2*∆ mutants were in bold. *ctr4*^+^ , a potential target of Set3C, is in red. RNA levels of *dh*, *dg* and *otr1*::*ura4*^+^ are extracted from the raw data manually (in blue), where *p* and *q* value is not available.

**Supplementary Table S2. List of strains used in this study.**

| **Strain** | **Genotype** | **Source** |
| --- | --- | --- |
| FY648 | *h^+^,leu1-32,ade6-m210,ura4-DS/E,otr1R(SphI)::ura4^+^* | Robin Allshire^1^ |
| LHP209 | *h^+^,leu1-32,ade6-m210,ura4-DS/E,otr1R(SphI)::ura4^+^, set3*Δ*::hphMX6* | This study |
| LHP208 | *h^+^,leu1-32,ade6-m210,ura4-DS/E,otr1R(SphI)::ura4^+^, hos2*Δ*::hphMX6* | This study |
| LHP207 | *h^+^,leu1-32,ade6-m210,ura4-DS/E,otr1R(SphI)::ura4^+^, snt1*Δ*::hphMX6* | This study |
| LHP206 | *h^+^,leu1-32,ade6-m210,ura4-DS/E,otr1R(SphI)::ura4^+^, hif2*Δ*::hphMX6* | This study |
| LHP306 | *h^+^,leu1-32,ade6-m210,ura4-DS/E,otr1R(SphI)::ura4^+^, dcr1*Δ*::hphMX6* | This study |
| LHP375 | *h^+^,leu1-32,ade6-m210,ura4-DS/E,otr1R(SphI)::ura4^+^, ago1*Δ*::hphMX6* | This study |
| LHP376 | *h^+^,leu1-32,ade6-m210,ura4-DS/E,otr1R(SphI)::ura4^+^,set3-3FLAG::kanMX6* | This study |
| LHP378 | *h^+^,leu1-32,ade6-m210,ura4-DS/E,otr1R(SphI)::ura4^+^,set3-FLAG::kanMX6,hos2*Δ*::hphMX6* | This study |
| LHP379 | *h^+^,leu1-32,ade6-m210,ura4-DS/E,otr1R(SphI)::ura4^+^, set3ΔPHD-3FLAG::kanMX6* | This study |
| LHP380 | *h^+^,leu1-32,ade6-m210,ura4-DS/E,otr1R(SphI)::ura4^+^, set3ΔSET-3FLAG::kanMX6* | This study |
| LHP381 | *h^+^,leu1-32,ade6-m210,ura4-DS/E,otr1R(SphI)::ura4^+^,hos2-3FLAG::kanMX6* | This study |
| LHP382 | *h^+^,leu1-32,ade6-m210,ura4-DS/E,otr1R(SphI)::ura4^+^,hos2-3FLAG::kanMX6,set3*Δ*::hphMX6* | This study |
| LHP383 | *h^+^,leu1-32,ade6-m210,ura4-DS/E,otr1R(SphI)::ura4^+^,rik1-3FLAG::kanMX6* | This study |
| LHP384 | *h^+^,leu1-32,ade6-m210,ura4-DS/E,otr1R(SphI)::ura4^+^,rik1-3FLAG::kanMX6,set3*Δ*::hphMX6* | This study |
| LHP385 | *h^+^,leu1-32,ade6-m210,ura4-DS/E,otr1R(SphI)::ura4^+^,rik1-3FLAG::kanMX6,hos2*Δ*::hphMX6* | This study |
| SPJ390 | *h^+^,3FLAG-clr4 otr1R::ura4+ ade6-M216 leu1-32 ura4-DS/E his2* | Shiv Grewal^2^ |
| LHP386 | *h^+^,3FLAG-clr4 otr1R::ura4+ ade6-M216 leu1-32 ura4-DS/E his2, set3*Δ*::hphMX6* | This study |
| LHP387 | *h^+^,3FLAG-clr4 otr1R::ura4+ ade6-M216 leu1-32 ura4-DS/E his2, hos2*Δ*::hphMX6* | This study |

Reference:

[1] Allshire RC, Nimmo ER, Ekwall K, Javerzat JP, Cranston G (1995) Mutations derepressing silent centromeric domains in fission yeast disrupt chromosome segregation. *Genes Dev.* 9: 218-233

[2] Jia S, Kobayashi R, Grewal SI. (2005) Ubiquitin ligase component Cul4 associates with Clr4 histone methyltransferase to assemble heterochromatin. *Nat Cell Biol.* 7(10):1007-13.

**Supplementary Table S3. Primers used in Real-Time PCR and siRNA analysis**

| **Primer name** | **Sequence** | **Used for** |
| --- | --- | --- |
| *act1^+^* forward | AACCCTCAGCTTTGGGTCTT | RT-PCR |
| *act1^+^* reverse | TTTGCATACGATCGGCAATA | RT-PCR |
| *otr dh* forward | ATGAAATCGTTTACCGCTTCTCC | ChIP, RT-PCR |
| otr *dh* reverse | TTGAAGATGGCGTATGTAGTGCT | ChIP, RT-PCR |
| otr *dg* forward | CCATCACCACTTTCATCTCC | ChIP, RT-PCR |
| otr *dg* reverse | CAGGATACCTAGACGCACAA | ChIP, RT-PCR |
| *otrR::ura4*^+^ forward | GAATGGTTTGAGAAGCATACC | ChIP, RT-PCR |
| *otrR::ura4*^+^ reverse | GAGTACGATATTGCTGTCCC | ChIP, RT-PCR |
| *fbp1*^+^ forward | GTCGAACGGATGCTGCAAAC | ChIP |
| *fbp1*^+^ reverse | GGTACCTACACTAACACCGG | ChIP |
| *cen* probe1 forward | AGTCAACTGAACAACGCATCTAC | Northern |
| *cen* probe1 reverse | AACTCCTGCTTATCGTCTTCTTT | Northern |
| *cen* probe2 forward | ATCTGCCATCACTTTATTTCTCC | Northern |
| *cen* probe2 reverse | TCAACCTTCCGACGCAAATCACC | Northern |
| *cen* probe3 forward | AATATGCTGCGGTTCACCCTTAA | Northern |
| *cen* probe3 reverse | TAGCCATTTGCTTAACTTACTGTCTCA | Northern |
| *cen* probe4 forward | TCATCAGCCTCTCTCTATATCTCTA | Northern |
| *cen* probe4 reverse | GACAGAATGGATGGATATTGACAG | Northern |
| *snoRNA U24* probe | GATTTGTTTTGTCTCATCGAGCC | Northern |
| *atl1*^+^ forward | ATATCTCGCGGATCCAATGGCT | ChIP, RT-PCR |
| *atl1*^+^ reverse | CTCTCAATTCGTACTGCACCATC | ChIP, RT-PCR |
| *adh4*^+^ forward | GCGCCGCAAATGCCAAACAATG | ChIP, RT-PCR |
| *adh4*^+^ reverse | ATCACTGCGGCTCTGTCTCCAA | ChIP, RT-PCR |
| *rdp1*^+^ forward | CCACATCAGCCGACAAGTTTTCTCT | RT-PCR |
| *rdp1*^+^ reverse | TCTTTCATCATAAACTTGCCCGAGC | RT-PCR |
| *hrr1*^+^ forward | TGGGAAATGGTGAACAAAAATACAG | RT-PCR |
| *hrr1*^+^ reverse | GAAACTCGTAGTATTATTATCGGCG | RT-PCR |
| *cid12*^+^ forward | ATGAAGAAGGTTTGAGTGATAATGC | RT-PCR |
| *cid12*^+^ reverse | ATCACTTATAGATAATGTTGTTCCA | RT-PCR |
| *arb1*^+^ forward | CGGTAATAGAAGAAAAAAGAGGGTA | RT-PCR |
| *arb1*^+^ reverse | TTCTCACCTGCTGTATAATGTCTCA | RT-PCR |
| *arb2*^+^ forward | ATTACCAACGATTTTTACTTGAGGA | RT-PCR |
| *arb2*^+^ reverse | AATGATTACCCTACCAAGTACAGCA | RT-PCR |
| *ago1*^+^ forward | ATATCTTCGAGTGCATTTTGGCGTA | RT-PCR |
| *ago1*^+^ reverse | CAGGTCAGTACCGACATTATTGCGA | RT-PCR |
| *tas3*^+^ forward | CTGGAGTCACAAAGGAGGTAAGAGC | RT-PCR |
| *tas3*^+^ reverse | ACTGGCTTCTCCTTTCCATCCGTAA | RT-PCR |
| *chp1*^+^ forward | AGCCTGCCCGAAGGATTGTA | RT-PCR |
| *chp1*^+^ reverse | AAGTAGAAAGGATTAACATCAGGGC | RT-PCR |
| *dcr1*^+^ forward | GGAGGCAAAAAGATACCCTTCATAA | RT-PCR |
| *dcr1*^+^ reverse | TTTGAAGCCATTGCTCTCGCTCTAC | RT-PCR |
| *clr4*^+^ forward (ORF) | GATACTTGGGAGCCCCCTGAGAACC | ChIP, RT-PCR |
| *clr4*^+^ reverse (ORF) | TTGATGTTGGTGCTTTTGTCTACTG | ChIP, RT-PCR |
| *rik1*^+^ forward (ORF) | AAGACTACCAATCCCACCTTTCGAC | ChIP, RT-PCR |
| *rik1*^+^ reverse (ORF) | TGGGTCCTAAGTTGGGTATTGAGTC | ChIP, RT-PCR |
| *raf1*^+^ forward | GGTGAAAAGAAGAACGGATACGCAA | RT-PCR |
| *raf1*^+^ reverse | GATAATGACGGTTTCGGGCAAGGTA | RT-PCR |
| *raf2*^+^ forward | TGCGTTACAAATAAAGCAGGGGAAT | RT-PCR |
| *raf2*^+^ reverse | TCCCAGAACAATGCTCTCATCAAAC | RT-PCR |
| *pcu4*^+^ forward | CAGGTTACCAAGAGTTATATTCGGG | RT-PCR |
| *pcu4*^+^ reverse | TTAGTGTCTGTTTCAGTCCCTTCAA | RT-PCR |
| *ctr4*^+^ forward (ORF) | AACCGCAAAGGCTTCTTCTTGTAAA | ChIP, RT-PCR |
| *ctr4*^+^ reverse (ORF) | AGAAAATCTACGGACACACCAACGA | ChIP, RT-PCR |
| *clr4*^+^ forward (promoter) | CTGTTGTTTCATTTCTTTTATTATT | ChIP, RT-PCR |
| *clr4*^+^ reverse (promoter) | TTACCGCAGTTCTATGTTATCGTTA | ChIP, RT-PCR |
| *rik1*^+^ forward (promoter) | TGCTAGACATCCGTAGGTTTTCATT | ChIP, RT-PCR |
| *rik1*^+^ reverse (promoter) | TATTGAAAACAAGAACTAGGCACGC | ChIP, RT-PCR |
| *ctr4*^+^ forward (promoter) | GAGACGATTGTTTGACTGAAAGGCT | ChIP, RT-PCR |
| *ctr4*^+^ reverse (promoter) | ATCAATGGGCAGAATCTCAACGAAT | ChIP, RT-PCR |
| *tlh1^+^* forward | GGCGAATGTG TATGTTGTGC ATC | ChIP, RT-PCR |
| *tlh1^+^* reverse | GTTTCTGGCA ATGTCGGTTT CAC | ChIP, RT-PCR |
| *cenH* forward | CCATAGTAGT ATGGCTATGA ATGGAAG | ChIP, RT-PCR |
| *cenH* reverse | TTCAAGTCTT CTTTATACGT TTGCATG | ChIP, RT-PCR |
